# Parameters affecting baseline hip function in patients with cam-derived femoroacetabular impingement syndrome: data analysis from the German Cartilage Registry

**DOI:** 10.1186/s10195-021-00596-6

**Published:** 2021-08-04

**Authors:** Sebastian Serong, Moritz Schutzbach, Stefan Fickert, Philipp Niemeyer, Christian Sobau, Gunther Spahn, Wolfgang Zinser, Stefan Landgraeber

**Affiliations:** 1 Department of Orthopaedics & Orthopaedic Surgery, Saarland University Medical Centre, Kirrberger Strasse 100, 66421 Homburg, Germany; 2 Department of Orthopaedics & Traumatology, University of Duisburg-Essen, Essen, Germany; 3 Sporthopaedicum Straubing, Straubing, Germany; 4 Department of Orthopaedic Surgery and Traumatology, Mannheim University Hospital, Mannheim, Germany; 5 OCM Clinic, Munich, Germany; 6 Department of Orthopaedics and Trauma Surgery, Freiburg University Hospital, Freiburg im Breisgau, Germany; 7 ARCUS Sports Clinic, Pforzheim, Germany; 8 Center of Trauma and Orthopaedic Surgery and Jena University Hospital, Jena, Germany; 9 Department of Orthopaedic Surgery and Traumatology, St. Vinzenz-Hospital Dinslaken, Dinslaken, Germany

**Keywords:** Femoroacetabular impingement syndrome, Cam morphology, iHOT-33, Baseline data, Patient-reported outcome

## Abstract

**Background:**

Using the database of the German Cartilage Registry (KnorpelRegister DGOU), this study aims to present patient- and joint-related baseline data in a large cohort of patients with cam-derived femoroacetabular impingement syndrome (FAI) and to detect symptom-determining factors.

**Materials and methods:**

Requiring cam morphology as the primary pathology, 362 patients were found to be eligible for inclusion in the study. The assessment of preoperative baseline data was performed using the patient-reported outcome measure—International Hip Outcome Tool (iHOT-33). Descriptive statistics were performed to present baseline data. Univariate and multiple regression with post hoc testing were used to identify patient- and joint-related factors that might affect the preoperative iHOT-33 and its subscores, respectively.

**Results:**

The study collective’s mean age was 36.71 ± 10.89 years, with 246 (68%) of them being male. The preoperative mean iHOT-33 total was 46.31 ± 20.33 with the subsection “sports and recreational activities” presenting the strongest decline (26.49 ± 20.68). The parameters “age,” “sex,” “body mass index” (BMI), and the confirmation of “previous surgery on the affected hip” were identified to statistically affect the preoperative iHOT-33. In fact, a significantly lower mean baseline score was found in patients aged > 40 years (*p* < 0.001), female sex (*p* < 0.001), BMI ≥ 25 kg/m^2^ (*p* = 0.002) and in patients with previous surgery on the affected hip (*p* = 0.022). In contrast, the parameters defect grade and size, labral tears, and symptom duration delivered no significant results.

**Conclusions:**

A distinct reduction in the baseline iHOT-33, with mean total scores being more than halved, was revealed. The parameters “age > 40 years,” “female sex,” “BMI ≥ 25,” and confirmation of “previous surgery on the affected hip” were detected as significantly associated with decreased preoperative iHOT-33 scores. These results help to identify symptom-defining baseline characteristics of cam-derived FAI syndrome.

*Trial registration*: The German Cartilage Registry is conducted in accordance with the Declaration of Helsinki and registered at germanctr.de (DRKS00005617). Registered 3 January 2014—retrospectively registered. The registration of data was approved by the local ethics committees of every participating institution. Primary approval was given by the ethics committee at the University of Freiburg (No. 105/13). https://www.drks.de/drks_web/navigate.do?navigationId=trial.HTML&TRIAL_ID=DRKS00005617

## Introduction

Over the last two decades, cam morphology and the associated femoroacetabular impingement syndrome (FAI) have been identified as a common source of hip pain and premature cartilage damage [1–4]. Although its pathogenesis is not yet fully understood, considerable progress has been made regarding various factors surrounding this pre-arthritic bony malformation. It is known that cam-type FAI predominantly affects young and active males, especially those participating in high-level impact sports [5–8]. In these patients, alterations in the capital femoral epiphysis, presumably a response to repetitive sports-related microtrauma, are regularly detected [9, 10]. Due to the pathological anatomy of the head–neck junction, cam-derived cartilage defects are typically found on the acetabular side of the joint [11, 12]. In this connection, labral tears are a further common finding in patients with cam morphology [13, 14].

The symptoms commonly described in cases of symptomatic FAI include motion-related or position-related pain in the hip and groin and functional limitations such as a reduced range of motion and even alterations in gait parameters [7, 15, 16]. For better evaluation of hip function in general, the patient-reported International Hip Outcome Tool (iHOT-33) was introduced in 2012. The iHOT-33 is a self-administered evaluative questionnaire to measure health-related quality of life in individuals with hip disorders [17]. Among other patient-reported outcome measures (PROMs), it has been proven to be reliable and highly responsive to clinical change and is therefore used regularly worldwide [18–22]. The iHOT-33 comprises 33 items that are categorized into four thematic sections: “symptoms and functional limitations,” “sports and recreational activities,” “job-related concerns,” and “social, emotional and lifestyle concerns.”

To reliably evaluate pre- and postoperative hip function in patients with chondral defects due to multiple causes, the iHOT-33 is also used in the German Cartilage Registry (KnorpelRegister DGOU). This multicenter, nationwide register aims at collecting baseline and follow-up data in connection with the treatment of osteochondral defects of the hip, as well as the knee and the ankle joint.

Concerning the parameters affecting hip function in patients with symptomatic FAI in general and with cam-derived FAI in particular, studies usually focus on the assessment of therapy outcome. In these studies, several factors, such as age, sex, or body mass index (BMI), that influence treatment results have so far been identified [23–26]. However, little is known about the factors that determine preoperative symptoms. It is to be hypothesized that parameters found to be influential on the treatment outcome also determine functional limitations preoperatively. Therefore, this study’s purpose is to present patient- and joint-related baseline data in a large cohort of patients with symptomatic femoroacetabular impingement syndrome (FAI) due to cam morphology and to detect symptom-determining factors.

## Materials and methods

Data for the present study were taken from the German Cartilage Registry (KnorpelRegister DGOU). The German Cartilage Registry is a multicenter register with over 70 participating clinics that was set up in 2013 to evaluate the long-term outcomes of different methods of surgical cartilage repair on the hip, knee, and ankle joint. The registry is conducted in accordance with the Declaration of Helsinki and was registered in the German Clinical Trials Register. Approval was received from each ethics committee before the enrollment of the first patients. The participants were generally included prior to surgery with informed consent given by every patient. Data collection was performed via web-based input.

**Fig. 1:**
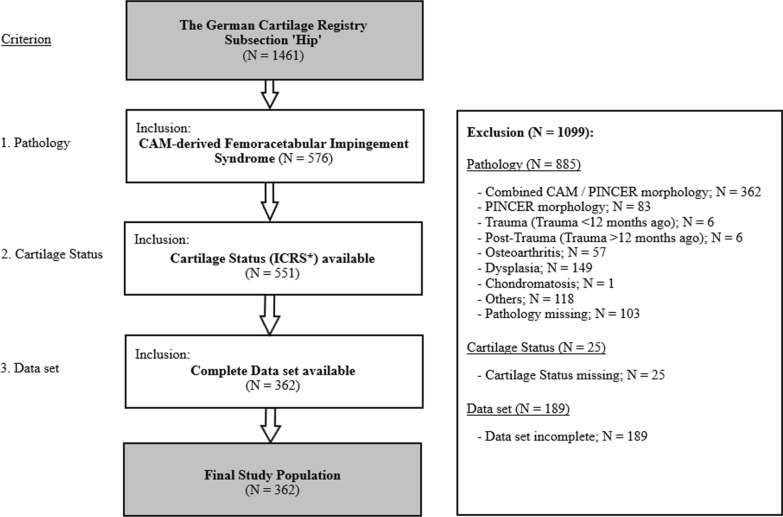
Selection procedure for the study group on the basis of the subsection “hip” of the German Cartilage Registry; ICRS*–International Cartilage Repair Society

Up to August 2019, the registry’s subsection “hip” contained data from 1461 patients that underwent hip surgery, particularly for reasons of cartilage damage. To solely assess patients with cam-derived FAI, only patients with documentation of isolated cam morphology that was declared causal of cartilage damage were included. Patients with record of other or multiple hip pathologies were excluded. With regard to FAI in particular, cases of isolated Pincer- or combined Cam-/Pincer morphology were excluded as well, though, in cases of isolated cam morphology, cartilage damage and labral tears were judged as cam-derived and consequently kept for evaluation. Further exclusion was made on the basis of missing documentation of the cartilage status. Consequently, 551 patients were generally considered eligible for inclusion. However, as several entries presented with incomplete datasets, these were excluded as well, ultimately resulting in 362 patients to form the final study group. Detailed information on the inclusion/exclusion procedure is provided in Fig. 1. A sample size of 362 produces a two-sided 95% confidence interval with a distance from the mean to the limits that is equal to 2.6 when the estimated standard deviation of preoperative iHOT-33 total is 25 points.

General patient data as well as pre- and postoperative data collection was performed via web-based remote data entry system. Perioperative data, including defect- and joint-specific characteristics, procedure-related information, and diagnosis confirmation, were provided by the treating surgeon on the basis of the intraoperative findings. Except one open procedure, all other study patients were treated by means of hip arthroscopy (*n* = 259) or arthroscopically assisted (*n* = 94). Overall hip function was measured using the iHOT-33 and its subsections. The total scores, as well as the subscores, were calculated as a mean of the specific item responses ranging from 0 to 100, with 100 representing the best possible quality-of-life score. Only preoperative baseline data were used in this analysis.

Statistical analysis was performed using SPSS Statistics (Version 21.0.0.0, IBM). Descriptive data are presented as mean (± standard deviation (SD) and 95% confidence intervals (CI)), percentage of the total and in total numbers. To detect factors possibly affecting preoperative iHOT-33, univariate and multiple regression analysis with simultaneous entry was performed. Metric and dichotomous variables were directly entered in the regression model; categorical variables were dummy coded first. Post hoc Tukey’s test and Student’s *t*-test were used to evaluate the subgroups of statistically influencing variables. The two-sided level of significance was set at *p* ≤ 0.05.

## Results

### Baseline data

Sex distribution of the study group was nearly 2:1 (male versus female), with an overall mean age of 36.71 (± 10.89) years. The mean symptom duration at the date of surgery was 23.42 (± 23.02) months. Three patients (0.8%) presented with isolated femoral cartilage damage. All other study participants had isolated acetabular (*n* = 119/32.9%) or multilocular (*n* = 240/66.3%) cartilage lesions. Concomitant labral tears were found in 319 patients (88.1%). Detailed patient characteristics are presented in Table 1.

**Table 1:** Baseline data of the study group’s patient- and joint-specific characteristics

Parameter	Mean ± SD or *n* (%)
Age, years	36.71 ± 10.89
Sex, male/female	246 (68.0%)/116 (32.0%)
Smoking status, non-smoker/smoker/ex-smoker	274 (75.7%)/78 (21.5%)/10 (2.8%)
Body mass index, kg/m^2^	24.90 ± 3.61
Previous surgery on the affected hip, no/yes	336 (92.8%)/26 (7.2%)
Cartilage defect grade (ICRS*), I/II/IIIa and b/IVa and b	40 (11.0%)/103 (28.5%)/153 (42.3%)/66 (18.2%)
Cartilage defect size, mm^2^	157.76 ± 204.86
Labral tear (% of vertical diameter), none/ < 33/33–66/ > 66	43 (11.9%)/88 (24.3%)/96 (26.5%)/135 (37.3%)
Number of chondral defects, 1/2/3	313 (86.5%) / 37 (10.2%)/12 (3.3%)
Symptom duration, months	23.42 ± 23.02

*SD* standard deviation, ICRS* International Cartilage Repair Society

The study group’s preoperative iHOT-33 total was 46.31 (± 20.33). The corresponding subscores were also distinctly lowered, with the greatest decrease for the “sports and recreational activities” score **(**Table 2).

**Table 2:** Baseline data of the study group’s iHOT-33 total and subscores

International Hip Outcome Tool (iHOT-33)	Mean ± SD (95% CI)
Total	46.31 ± 20.33 (43.21–48.41)
Symptoms and functional limitations	54.73 ± 23.36 (52.31–57.14)
Sports and recreational activities	26.49 ± 20.68 (24.34–28.63)
Job-related concerns	49.37 ± 29.33 (45.98–52.76)
Social, emotional, and lifestyle concerns	43.33 ± 23.99 (40.85–45.81)

*SD* standard deviation, *CI* confidence interval

### Influencing factors

Univariate and multiple regression analysis were performed to detect symptom-influencing factors in preoperative patients with cam-type FAI syndrome. The variables included and the corresponding statistical results are listed in Table 3. Particularly, multiple regression revealed four factors to be statistically significantly associated with higher preoperative iHOT-33 scores: “younger patient age” (*p* < 0.001), “male sex” (*p* < 0.001), “lower BMI” (*p* = 0.030), and the absence of “previous surgeries on the affected hip” (*p* = 0.003). All other analyzed parameters were shown to be not significantly associated with certain tendencies in iHOT-33 scoring, among others, the cartilage parameters “defect size” (*p* = 0.622) and “defect grade” (*p* = 0.405), the presence of “labral tears” (*p* = 0.534), and the overall “symptom duration” (*p* = 0.279) (Table 3).

**Table 3:** Univariate and multiple regression of parameters with possible influence on baseline iHOT-33

Parameter	Baseline iHOT-33	
	Univariate regression	Multiple regression
Age	*p* < 0.001*** *β* = −0.293	*p* < 0.001*** *β* = −0.203
Sex (1 male, 2 female)	*p* < 0.001*** *β* = −0.293	*p* < 0.001*** *β* = −0.311
Smoking status	Not significant (*p* = 0.114)	Not significant (*p* = 0.122)
Body mass index	*p* = 0.023* *β* = −0.120	*p* = 0.030* *β* = −0.120
Previous surgery on the affected hip	*p* = 0.022* *β* = −0.121	*p* = 0.003** *β* = −0.151
Cartilage defect grade (ICRS*)	Not significant (*p* = 0.178)	Not significant (*p* = 0.405)
Cartilage defect size	Not significant (*p* = 0.949)	Not significant (*p* = 0.622)
Labral tear	Not significant (*p* = 0.893)	Not significant (*p* = 0.534)
Number of defects	Not significant (*p* = 0.787)	Not significant (*p* = 0.125)
Symptom duration	Not significant (*p* = 0.206)	Not significant (*p* = 0.279)

ICRS* International Cartilage Repair Society, *p* *p* value, *β* regression coefficient

*p ≤ 0.05, **p ≤ 0.01, ***p ≤ 0.001

**Fig. 2:**
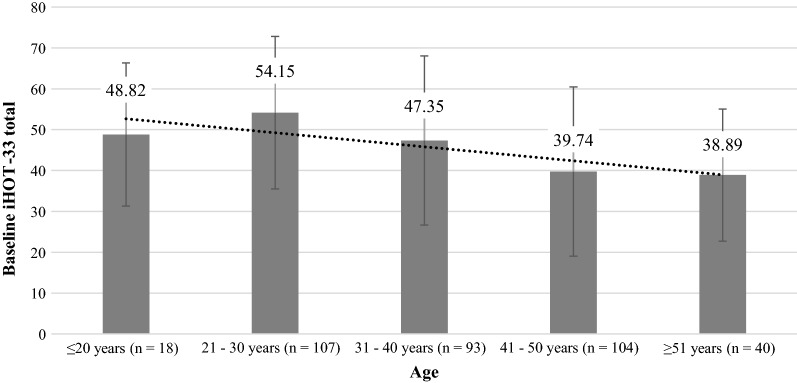
Age-dependent decrease of mean baseline score of the 33-item International Hip Outcome Tool (iHOT-33)

Descriptive statistics for the parameter “age” revealed that increasing patient age almost constantly decreases the mean baseline iHOT-33 (Fig. 2). Here, the age of 40 years sets a distinct limit, with younger patients having a significantly better iHOT-33 than older patients (50.81 (± 19.65) versus 39.50 (± 19.49), *p* < 0.001).

Analysis of the parameter “sex” showed males to have a mean preoperative iHOT-33 of 50.61 (± 18.78) compared with 37.20 (± 20.55) for females (*p* < 0.001). For “age” and “sex,” results showed a similar pattern for the according subscores except for “sports and recreational activities.” As lower BMI was revealed to be positively associated with higher iHOT-33 scores, the study collective was subdivided according to the categories proposed by the World Health Organization (WHO). Study patients with a BMI below pre-obesity (< 25 kg/m^2^) were proven to have significantly better baseline iHOT scores than those with a BMI ≥ 25 kg/m^2^ (49.26 ± 19.67 versus 42.72 ± 20.59, *p* = 0.002). Regarding “previous surgery on the affected hip,” patients without previous operations had a mean preoperative iHOT-33 of 46.99 (± 20.26), whereas those with previous hip surgery in their medical history had a significantly lower mean score of 37.51 (± 19.46; *p* = 0.022). Detailed statistics of the affecting parameters are presented in Table 4.

**Table 4:** Parameter-dependent differences in iHOT-33 total and subscores with statistical analysis using post hoc Tukey’s and Student’s *t* test

Parameter	Baseline iHOT-33				
	Total	Symptoms and functional limitations	Sports and recreational activities	Job-related concerns	Social, emotional, and lifestyle concerns
	Mean ± SD (95% CI)				
Age					
≤ 40 years	50.81 ± 19.65 (48.19–53.43)	60.62 ± 22.33 (57.64–63.60)	27.18 ± 21.11 (24.36–30.00)	56.92 ± 28.66 (52.66–61.19)	46.96 ± 23.29 (43.85–50.07)
> 40 years	39.50 ± 19.49 (36.29–42.71)	45.80 ± 22.09 (42.16–49.44)	25.42 ± 20.02 (22.10–28.75)	37.72 ± 26.49 (32.80–42.63)	37.82 ± 24.07 (33.86–41.78)
	*p* < 0.001***	*p* < 0.001***	*p* = 0.432	*p* < 0.001***	*p* < 0.001***
Sex					
Male	50.61 ± 18.78 (48.25–52.97)	60.35 ± 21.00 (57.71–62.98)	27.77 ± 21.46 (25.06–30.47)	54.11 ± 28.72 (50.07–58.16)	47.05 ± 23.49 (44.10–50.00)
Female	37.20 ± 20.55 (33.42–40.98)	42.81 ± 23.74 (38.44–47.17)	23.80 ± 18.72 (20.35–27.24)	39.49 ± 28.24 (33.71–45.28)	35.43 ± 23.21 (31.16–39.70)
	*p* < 0.001***	*p* < 0.001***	*p* = 0.074	*p* < 0.001***	*p* < 0.001***
Body mass index					
< 25 kg/m^2^	49.26 ± 19.67 (46.51–52.01)	58.75 ± 22.64 (55.58–61.91)	27.57 ± 19.90 (24.79–30.36)	53.57 ± 28.31 (49.16–57.98)	44.71 ± 23.06 (41.49–47.94)
≥ 25 kg/m^2^	42.72 ± 20.59 (39.53–45.90)	49.82 ± 23.36 (46.20–53.43)	25.14 ± 21.59 (21.78–28.50)	44.13 ± 29.83 (38.94–49.33)	41.63 ± 25.06 (37.76–45.51)
	*p* = 0.002**	*p* < 0.001***	*p* = 0.268	*p* = 0.006**	*p* = 0.224
Previous surgery					
No	46.99 ± 20.26 (44.82–49.17)	55.56 ± 23.14 (53.07–58.04)	26.79 ± 21.05 (24.53–29.07)	49.40 ± 29.53 (45.87–52.93)	44.05 ± 24.05 (41.47–46.63)
Yes	37.51 ± 19.46 (29.65–45.37)	43.97 ± 24.01 (34.27–53.67)	22.59 ± 14.92 (16.56–28.61)	48.99 ± 27.05 (35.95–62.03)	33.91 ± 21.58 (25.20–42.63)
	*p* = 0.022*	*p* = 0.015*	*p* = 0.191	*p* = 0.950	*p* = 0.029*

*SD* standard deviation, *CI* confidence interval, *p* *p* value

*p ≤ 0.05, **p ≤ 0.01, ***p ≤ 0.001

## Discussion

This study’s results demonstrate the distinct reduction of function-related parameters due to cam-derived FAI syndrome. In particular, the parameters “age > 40 years,” “female sex,” “BMI > 25,” and “previous surgery on the affected hip” were identified as associated with statistically significantly lowered scores in preoperative baseline iHOT-33.

With “100” representing the best possible quality-of-life score, the mean iHOT-33 in the study cohort was less than half, indicating the severity of FAI-associated health restrictions. Even greater deteriorations were found for the sports and recreational activities subscore. These findings generally correlate with those in the current literature. However, this study’s baseline iHOT tends to be higher when compared with Nwachukwu et al., who reported preoperative iHOT scores of 39.7 and 40.6, respectively in own FAI collectives [27, 28]. Griffin et al. reported an even lower baseline iHOT-33 of 35.6 when assessing patients with symptomatic FAI prior to conservative treatment [29]. With regard to other PROMS, reported preoperative and “preconservative” values of the Hip Outcome Score–Activities of Daily Living (HOS-ADL) tend to be higher, with results ranging from 65.7 to 73.9 [26–28, 30], though the distinct baseline reduction of the sport-specific subscale (HOS-SSS) in comparison with the HOS-ADL confirms this study’s findings regarding the poor sports-specific iHOT subscores [26–28]. To appropriately interpret this finding, one has to consider the Patient Acceptable Symptomatic State (PASS) related to hip arthroscopy and FAI. In this context, Maxwell et al. reported a PASS score of 58 points for the iHOT-33 at 2 years follow-up after hip arthroscopy [31]. Using the 12-item iHOT, Robinson et al. recently reported a PASS threshold of 59.5 points following arthroscopy for FAI in a UK population [32]. So, as both identified PASS scores were distinctly undercut by the study group’s mean preoperative score, this additionally underlines the clinical relevance of functional limitations in this context. However, one also has to be aware that these limitations could not only be cam-derived as concomitant cartilage and labrum defects might have contributed as well. As concerns the iHOT-33 associated subscores, all except “symptoms and functional limitations” were reduced by more than half as well. The greatest reductions were seen for “sports and recreational activities.” However, this is not surprising as the evaluated patient collective was rather young. It appears likely that sports activities play an important role in their everyday lives and that, therefore, their general level of activity is higher compared with older people [33, 34]. In this context, it was Agricola et al. who described the development of cam morphology in adolescent athletes before growth plate closure [5, 35]. Further confirmation of this concept was provided by Palmer et al., who also depicted the underlying causality with physical activities and particularly emphasized the risk of developing secondary hip pathology after the occurrence of cam deformity [36]. Altogether, it can be reasonably assumed that, due to the combination of younger patients’ age and the performance of sports activities, functional limitations become especially apparent in the iHOT’s sports-specific subscore and are accompanied by a relevant reduction in the overall quality of life [37]. Moreover, not only the confirmation of sports activities alone but rather the level of its performance appears to be of importance. Compared with recreational athletes, high-level athletes have a higher likelihood of undergoing bilateral surgery in the case of symptomatic FAI [38].

In total, this study’s results were generally to be expected since the symptoms related to FAI are well known. Nevertheless, publications assessing preoperative baseline-data in depth are rare. This study’s findings enable quantification and identification of the core areas of preoperative functional limitations in cam-type FAI using the standardized and reliable iHOT-33 [17]. Furthermore, through the use of register data, the findings are based on a large, multicenter patient collective.

This study’s second objective was to identify factors that may influence preoperative hip function in patients with FAI due to cam morphology. Again, the iHOT-33 was taken as a measure for quantification of joint-related functional restrictions. Using univariate and multiple regression analysis, four factors were detected to be significantly correlated to the preoperative iHOT-33 score. These were the parameters “age,” “sex,” “body mass index,” and the confirmation of “previous surgeries on the affected joint.” In fact, the factors younger age, male sex, BMI < 25 kg/m^2^, and the absence of previous surgical treatment delivered significantly better results regarding the preoperative iHOT-33 scores. Interestingly, except for joint-related previous surgeries, these parameters were exactly the same as those recently identified by Sogbein et al., which confirms the initially proposed hypothesis. In their review on the predictors of outcome after arthroscopy for FAI, the above parameters were explicitly declared as predictors of a positive outcome [23]. Furthermore, their reported outcome-dependent thresholds concerning the patients’ age (< 45 years) and the BMI (< 24.5 kg/m^2^) correspond very closely with this study’s findings as ages < 40 years and BMI < 25 kg/m^2^ had a significantly better baseline iHOT-33 total. Apart from this, there are further publications that indicate the influential role of patients’ sex, age, and BMI on FAI-related treatment outcome [26, 39–41]. This suggests that there is a considerable overlapping of factors influencing postoperative results and those affecting preoperative function and symptoms in the FAI syndrome. This is of relevance insofar as the majority of publications focus on the presentation of outcome data to evaluate treatment success [23–26, 39–41]. As mentioned above, little has been published on baseline data. But these data might help physicians gain a better understanding of the pathology of FAI and particularly the underlying cam morphology as a whole. In this context, this study’s results are supported by the findings of Nwachukwu et al., who also detected lower preoperative PROMs and patient-reported outcomes measurement information system scores (PROMIS) for the female sex and a higher BMI [42]. However, as statistical findings are not necessarily associated with clinical relevance, it was also Nwachukwu et al. who identified the iHOT-33’s minimal clinically important difference (MCID) after arthroscopic treatment of FAI syndrome at 12.1. [28] In light of that and with regard to the iHOT-33 total, only the sex-dependent preoperative mean difference was shown to exceed this threshold, though the age-dependent mean differences only narrowly missed passing the MCID.

As concerns the factors identified to possibly influence the baseline iHOT, it is known that the ageing of organisms is accompanied by an ongoing reduction in the ability of tissues to maintain themselves [43]. Obviously, this also affects joint-related tissue such as bone and cartilage where the deviant behavior of chondrocytes during the ageing process has been proven [44]. Therefore, it is comprehensible that increasing age is accompanied by lower functional scores pre- and postoperatively [13, 23, 26, 39, 40]. In this context, 40–45 years of age seem to mark a threshold [23]. With regard to sex as an affecting parameter, its role is not yet fully understood. Sogbein et al. postulated a possibly underlying laxity in female soft tissue that might affect postoperative results [23]. As this is a permanent state, it could affect preoperative function and symptoms as well. However, this is highly speculative and needs to be further investigated. Body mass index has been controversially discussed concerning its role in hip arthroscopy and FAI syndrome. Several publications have reported the negative impact of obesity on the outcome of arthroscopic treatment in general [45, 46]. Gupta et al. found lower patient-reported outcome scores in obese patients after hip arthroscopy specifically. They reported that these results were additionally accompanied by distinctly reduced preoperative scores, which in turn support this study’s findings [47, 48]. Considering a large number of previous publications on this aspect, Saltzman et al. concluded that, despite clinical differences, there are no definite associations between BMI and outcome parameters owing to a variety of confounding variables [41]. Therefore, the role of BMI in the evaluation of baseline data needs to be investigated further. This study’s fourth parameter associated with a significant reduction in the baseline iHOT-33 was the confirmation of previous surgeries on the affected hip. This is understandable as any surgical procedure has a certain failure rate that affects both the postoperative outcome and the preoperative scores in cases where revision surgery is performed. The statistical insignificance of the factors “defect grade,” “defect size,” “labral tears,” and “symptom duration” was rather surprising. Here, outcome studies indicated further influence on the postoperative results, which we expected would be confirmed by our baseline analysis as well [13, 23, 39, 40, 49].

The findings of this study have to be seen in the light of some limitations. First, the study only assesses patients with cam-type FAI. Results might have been different for FAI patients in general including Pincer and combined Cam + Pincer morphologies. Then, as the used registry focuses on cartilage therapy, all patients were characterized by concomitant chondral defects. Again, results might have been different for patients with cam morphology in absence of cartilage damage. As patients with “previous surgery on the affected hip” were included in the statistical analysis, it can be questioned whether their data should be used when assessing baseline data. However, as none of the statistics were substantially changed hereby and as this aspect is of some importance, these patients were deliberately included in the study. Finally, the findings are based on the analysis of register data. Any parameters not evaluated (e.g., joint space or “Tönnies grade”) or not (sufficiently) recorded by the register might have been unintentionally ignored or misinterpreted. Selection bias and non-response to follow-up questionnaires might also have led to biased results [50, 51].

## Conclusion

The study revealed a distinct reduction in the baseline iHOT-33, with mean total scores being more than halved. The parameters “age > 40 years,” “female sex,” “BMI ≥ 25 kg/m^2^,” and the confirmation of “previous surgery on the affected hip” were detected as associated with significantly lowered preoperative iHOT-33 scores. These findings help to identify symptom-defining baseline characteristics of cam-derived FAI syndrome. Moreover, as these factors have previously been identified to be associated with poorer postoperative outcome, additional information on chances and expectations of surgical FAI treatment for physicians and patients is provided.

## Data Availability

The data that support the findings of this study are available from the corresponding author upon reasonable request.

## Abbreviations

FAI: Femoroacetabular impingement syndrome
iHOT-33: International Hip Outcome Tool (33 items)
BMI: Body mass index
PROM: Patient-reported outcome measure
ICRS: International Cartilage Repair Society
SD: Standard deviation
CI: Confidence interval
WHO: World Health Organization
PAS: Patient acceptable symptomatic state
PROMI: Patient-Reported Outcomes Measurement Information System
MCI: Minimal clinically important difference
